# Factors affecting perception and acceptance of colonoscopy in patients with inflammatory bowel disease

**DOI:** 10.1016/j.pmedr.2024.102951

**Published:** 2024-12-18

**Authors:** Chang-Hung Liao, Peng-Jen Chen, Yu-Lueng Shih, Wei-Kuo Chang, Tsai-Yuan Hsieh, Tien-Yu Huang

**Affiliations:** a Division of Gastroenterology, Department of Internal Medicine, Tri-Service General Hospital, National Defense Medical Center, Taipei, Taiwan; b Taiwan Society of Inflammatory Bowel Disease, Taipei, Taiwan; c Taiwan Association of Small Intestinal Disease, Taoyuan, Taiwan

**Keywords:** Bowel preparation, Colonoscopy, Examination interval, Inflammatory bowel disease, Patient perceptions, Surveillance colonoscopies

## Abstract

**Objective:**

The noncompliance rate with routine or surveillance colonoscopies is high, and the underlying reasons remain unverified among Asian patients with inflammatory bowel disease (IBD). This study aimed to examine the perceptions of Asian patients with IBD regarding bowel preparation and colonoscopy and their attitudes toward the recommended intervals for colonoscopies.

**Methods:**

Using data from one medical center between July 2020 and May 2022, we analyzed the perceptions of bowel preparation and colonoscopy and attitudes toward examination intervals among 94 patients with IBD (Crohn's disease, 41; ulcerative colitis, 53). The patients' perceptions of the four components associated with the colonoscopy procedure (embarrassment, pain, use of bowel-cleansing agents, and stress) were assessed via a questionnaire. Patients were asked to indicate the frequency at which they had scheduled colonoscopy and the frequency at which they desired to undergo the procedure.

**Results:**

“Bowel cleansing” and “pain” received the highest dissatisfaction rate. “Drink too much” was the greatest burden in bowel preparation. Younger age and younger age at diagnosis were associated with a greater burden of bowel preparation and pain. Younger patients and those diagnosed at an earlier age tended to prefer longer examination intervals.

**Conclusions:**

Bowel cleansing and abdominal pain were the most uncomfortable aspects associated with colonoscopy, especially when performed without sedation, among Asian patients with IBD. Younger patients and those with early diagnoses preferred longer examination intervals. Our findings can promote colonoscopy adherence and facilitate early detection of major complications in patients at high risk and those with long-term IBD.

## Highlights

• Patients experience discomfort and stress during bowel preparation.
• Younger patients and those with younger age at diagnosis preferred longer examination intervals.
• Discrepancy exists between patient-preferred and physician-recommended colonoscopy intervals.
• This is the first Asian study to explore perceptions of colonoscopy in patients with inflammatory bowel disease.

## 1 Introduction

Inflammatory bowel disease (IBD), including Crohn's disease and ulcerative colitis, is characterized by chronic gut inflammation that affects a patient's quality of life (Chang, 2020). Ileocolonoscopy or colonoscopy is well established as the mainstay procedure for diagnosing or monitoring the severity of IBD (Gomollon et al., 2017; Lichtenstein et al., 2018; Rubin et al., 2019). According to the STRIDE II consensus, endoscopic remission is a long-term target for IBD treatment to reduce the risk of complications, decrease relapse, and improve quality of life (Turner et al., 2021). Further, previous studies suggest that the cumulative extent of colonic inflammation and the duration of disease are risk factors for colitis-associated colorectal cancer (CAC) in patients with IBD (Shah and Itzkowitz, 2022; Murthy et al., 2021). A previous study reported that the cumulative probability of CAC in patients with ulcerative colitis was estimated to be 1.6 % after 10 years, 8.3 % after 20 years, and 18.4 % after 30 years of disease duration in patients with ulcerative colitis (Eaden et al., 2001). Long-term colonoscopic surveillance is necessary for patients with IBD to monitor the severity of mucosal inflammation and colitis-associated dysplasia or cancer (Shah and Itzkowitz, 2022; Murthy et al., 2021).

Regular colonoscopy is highly recommended for patients with IBD (Rubin et al., 2019; Murthy et al., 2021; Gordon et al., 2023). Despite these recommendations, colonoscopy has the lowest degree of acceptability among IBD monitoring tools (Buisson et al., 2017). In Western countries, colonoscopy is frequently performed under sedation to alleviate discomfort and enhance patient adherence. In contrast, routine colonoscopy without sedation is common in both the general population and patients with IBD in Asian countries, showing certain advantages and garnering preference in numerous cancer centers globally (Zhang et al., 2018). Consequently, pain during or after colonoscopy remains unavoidable in patients with IBD. Previous studies have found that patients with IBD experience more discomfort than the general population (Denters et al., 2013a). Adequate bowel preparation is required before a colonoscopy (Hassan et al., 2019; Millien and Mansour, 2020). Adequate bowel preparation is crucial for detecting colonic lesions and evaluating the mucosa (Martens and Bisschops, 2014). Patients with IBD have reported a significantly greater burden of preparation than other patient groups, including having to consume larger quantities of preparation agents and experiencing unpleasant tastes during the bowel preparation phase (Denters et al., 2013b; Ryhlander et al., 2019). Only 62 % of patients with IBD adhere to the recommended timing for colonoscopy; factors such as sex, age at diagnosis, medication use, and insurance coverage are associated with inconsistent adherence to the recommended timing for surveillance colonoscopies (Matthew et al., 2020).

The primary objective of this study was to examine the perceptions of Asian patients with IBD regarding bowel preparation and colonoscopy and their attitudes toward the recommended intervals for colonoscopies. The secondary aim was to compare the frequencies of physicians' recommendations and patient preferences to identify potential reasons for inconsistent adherence to surveillance colonoscopies among patients with IBD in Taiwan.

## 2 Methods

### 2.1 Study design

This study was conducted between July 2020 and May 2022 at the Tri-Service General Hospital in Taiwan. All patients diagnosed with IBD and scheduled for routine or surveillance colonoscopy, including those undergoing sedated colonoscopy, were invited to participate in this study. All participants underwent colonoscopy with carbon dioxide insufflation. The patients all signed an informed consent form before study inclusion. After providing consent, participants completed self-administered questionnaires following the colonoscopy; each patient completed the questionnaire within approximately 20–30 min. The study was approved by the Institutional Review Board of the Tri-Service General Hospital (approval No. B202005094).

### 2.2 Questionnaire design

The questionnaire was adapted from earlier studies (Denters et al., 2013a; Denters et al., 2013b; Friedman et al., 2013) and was first validated using healthcare workers (physicians and physician assistants) and then submitted to a test group of 10 patients with IBD to check for clarity. The questionnaire comprised three sections: (i) multiple choice questions for basic data and clinical characteristics; (ii) questions on the burden of colonoscopy with some questions scored using a 4-point Likert scale; and (iii) questions on the physician's recommendations regarding frequency of colonoscopy and patients' preferences.

### 2.3 Questionnaire for the burden of colonoscopy

This section was divided into four parts, namely “Embarrassment,” “Pain,” “Stress,” and “Burden of Drinking Bowel-Cleansing Agents.” Participants were instructed to utilize a 4-point Likert scale, with responses of 1 (“not at all”), 2 (“a little”), 3 (“quite”), and 4 (“very”). They were asked to rate the level of negative emotions associated with various elements of the procedure as follows:

1. Embarrassment/pain during bowel preparation
2. Embarrassment/pain/stress when the colonoscope was introduced (patients who selected sedated colonoscopy were excluded)
3. Embarrassment/pain/stress throughout the colonoscopy procedure (patients who underwent sedated colonoscopy were excluded)
4. Pain/stress after the examination
5. Stress while waiting for the final results
6. The burden associated with drinking bowel-cleansing agents (including aspects such as excessive consumption, unpleasant taste, and its impact on sleep)

### 2.4 Questionnaire for colonoscopy intervals

Participants were presented with two specific questions regarding their adherence to colonoscopy:

7. “According to your doctor's advice, how frequently should you undergo a colonoscopy?”
8. “Considering your physical condition and personal preferences, how often do you find it acceptable to undergo a colonoscopy?”

Participants were then provided with incremental time intervals and asked to indicate the frequency at which they had scheduled a colonoscopy, as well as the frequency they found acceptable for undergoing a colonoscopy.

### 2.5 Statistical analysis

The tests were two-sided, with a significance level (α) set at 0.05. Patient characteristics were presented as mean ± standard deviation for continuous variables and as the number of patients (%) for categorical variables. Differences between groups were assessed for statistical significance using analysis of variance or the Mann–Whitney *U* test for continuous variables, depending on the statistical distribution (normality was assessed using the Kolmogorov–Smirnov test). Chi-squared or Fisher's exact test was used for categorical data.

The assessment of patient adherence and negative emotions associated with various aspects of colonoscopy were analyzed using descriptive statistics. This involved summarizing the corresponding scores based on the proportion of participants who rated each item. Chi-squared or Fisher's exact test was used to determine any statistical relationship, specifically between one ordinal field and one nominal field or between two nominal fields. When statistically significant differences were observed, a correlation analysis was conducted, with the strength of the correlation indicated by the Phi/Cramer's V value for relationships involving ordinal and nominal variables. For the relationships between one ordinal field and one continuous field, statistical relationships were determined using Spearman's correlation coefficients. All statistical analyses were performed using IBM SPSS Statistics for Windows (ver. 21, IBM Corp., Armonk, N.Y., USA).

## 3 Results

The study included 94 patients: 53 (56.3 %) diagnosed with ulcerative colitis and 41 (43.6 %) diagnosed with Crohn's disease. Of the participants, 60 (63.8 %) were men, and 34 (36.2 %) were women. The average age of the participants was 46.2 ± 17.7 years, with a mean body mass index (BMI) of 21.9 ± 3.8 kg/m^2^. The average age at the time of diagnosis was 40.9 ± 17.4 years, and the mean duration of the disease was 5.1 ± 5.9 years. Notably, only eight patients (8.5 %) underwent bowel resection, all of whom had Crohn's disease. A summary of baseline characteristics is presented in Table 1.

**Table 1:** Clinical characteristics of patients with inflammatory bowel disease in Tri-Service General Hospital from 2020 to 2022 (*n* = 94).

	Total (n = 94)		Crohn's disease (*n* = 41)		UC (*n* = 53)		*p-value*⁎
Male, N (%)	60	(63.8)	26	(63.4)	34	(64.2)	0.941
Age, mean (years) (SD)	46.17	(17.7)	46.63	(18.2)	45.81	(17.6)	0.909
BMI, mean (kg/m^2^) (SD)	21.91	(3.7)	20.95	(3.8)	22.66	(3.6)	0.023
BMI Classification, N (%)							
Underweight (BMI < 18.5)	17	(18.1)	11	(26.8)	6	(11.3)	0.025
Normal range (18.5 ≤ BMI < 24)	52	(55.3)	23	(56.1)	29	(54.7)	
Pre-obesity (24 ≤ BMI < 27)	18	(19.1)	3	(7.3)	15	(28.3)	
Obese	7	(7.4)	4	(9.8)	3	(5.7)	
Education level, N (%)							
High school degree or below	34	(36.2)	15	(36.6)	19	(35.8)	0.941
College degree or above	60	(63.8)	26	(63.4)	34	(64.2)	
Age at Diagnosis, mean (years) (SD)	40.86	(17.4)	41.51	(18.5)	40.36	(16.7)	0.837
Disease duration, mean (years) (SD)	5.08	(5.92)	5.17	(6.88)	5.01	(5.13)	0.969
Disease duration, N (%)							
<10 years	80	(85.1)	36	(87.8)	44	(83.0)	0.572
≥10 years	14	(14.9)	5	(12.2)	9	(17.0)	
Previous bowel resection, N (%)	8	(8.5)	8	(19.5)	0	(0.0)	< 0.001
Previous of sedated colonoscopy, N (%)	46	(48.9)	24	(58.5)	22	(41.5)	0.101
Sedated colonoscopy chosen last time, N (%)	15	(16.0)	11	(26.8)	4	(7.5)	0.011
Smoking habits, N (%)							
Never	69	(73.4)	31	(75.6)	38	(71.7)	0.049
Quit smoking	19	(20.2)	5	(12.2)	14	(26.4)	
Social needs	3	(3.2)	2	(4.9)	1	(1.9)	
Smoker	3	(3.2)	3	(7.3)	0	(0.0)	
Drinking habits, N (%)							
Never	67	(71.3)	31	(75.6)	36	(67.9)	0.733
Quit drinking	8	(8.5)	2	(4.9)	6	(11.3)	
Social needs	15	(16.0)	6	(14.6)	9	(17.0)	
Drinker	4	(4.3)	2	(4.9)	2	(3.8)	
Exercise habits, N (%)							
None	13	(13.8)	8	(19.5)	5	(9.4)	0.186
Once or twice a week	18	(19.1)	5	(12.2)	13	(24.5)	
Three to five times a week	18	(19.1)	10	(24.4)	8	(15.1)	
Everyday	45	(47.9)	18	(43.9)	27	(50.9)	
Exercise time, N (%)							
Within 30 min	21	(40.4)	7	(28.0)	14	(51.9)	0.080
More than 30 min	31	(59.6)	18	(72.0)	13	(48.1)	
Western food, N (%)	27	(28.7)	12	(29.3)	15	(28.3)	0.918
UC, Location							
E1: Proctitis					30	(56.6)	
E2: Left side colitis					16	(30.2)	
E3: Extensive/Total colitis					7	(13.2)	
Crohn's disease, Age of diagnosis							
A1 ≤ 16 years			2	(4.9)			
A2 17–40 years			19	(46.3)			
A3 > 40 years			20	(48.8)			
Crohn's disease, Location							
L1: Ileal			8	(19.5)			
L2: colonic			12	(29.3)			
L3: Ileocolonic			21	(51.2)			
Crohn's disease, Behavior							
B1: Inflammatory			15	(36.6)			
B2: Stricturing			22	(53.7)			
B3: Penetrating			11	(26.8)			
P: Perianal			5	(12.2)			

SD, standard deviation; BMI, body mass index.

⁎ . *p*-values obtained via Chi-squared or Fisher's exact test.

### 3.1 Patients' feelings on the burden of colonoscopy

**Fig. 1:**
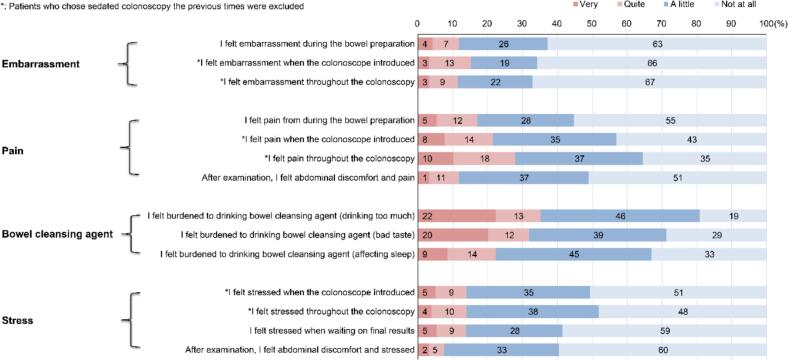
Percentage chart of feelings of patients with inflammatory bowel disease in Tri-Service General Hospital on colonoscopy burdens from 2020 to 2022.

Fig. 1 shows a percentage chart of personal feelings. Regarding embarrassment, 11 % of patients experienced significant embarrassment during bowel preparation for colonoscopy, while 16 % felt similarly embarrassed during the colonoscope introduction, which was also the most frequent answer to this question. Furthermore, 17 % of the patients regarded bowel preparation as quite or very painful, and 22 % of the patients felt that colonoscope insertion was quite or very painful (patients undergoing sedated colonoscopy were excluded). Many patients (28 %) experienced pain (patients who chose “quite” or “very” on this item) throughout the period of examination. Regarding bowel-cleansing agents, 44 % of patients felt uncomfortable, and the response of “felt like drinking too much” was the most frequent. A total of 35 % of patients felt quite or very uncomfortable with drinking too much. In addition, 32 % of the patients chose “quite” or “very” in response to the questions regarding a bad taste, and 23 % of patients also felt that their sleep was affected during the process of bowel preparation. Regarding stress, 14 % of patients felt quite or very stressed during colonoscopy or while waiting for the results, and 52 % of patients felt the most stressed during the examination, which was the most frequent response.

### 3.2 Correlation between each questionnaire item and patient characteristics

We further analyzed the correlation between each questionnaire item for the burden of colonoscopy and patient characteristics, and the results are shown in Table 2. Younger patients and patients diagnosed at an earlier age were more likely to feel embarrassment over “bowel preparation”,“ colonoscope introduced” and “during colonoscopy”. Additionally, patients presenting with a shorter disease duration had more painful sensations over “colonoscope introduced” and “during colonoscopy” (correlation coefficients of −0.225 and − 0.304, respectively). Younger patients and those diagnosed at an earlier age experienced more abdominal discomfort and pain after colonoscopy (correlation coefficients of −0.274 and − 0.234, respectively).

**Table 2:** Correlation matrix of personal feelings in patients with inflammatory bowel disease in Tri-Service General Hospital with important clinical parameters from 2020 to 2022.

	Sex	IBD type	BMI grouping		Sedation given	Education level	Disease duration grouping	Previous bowel resection	Age	Age at diagnosis	Disease duration	BMI	Smoking	Alcohol	Exercise	West food
	r	r	r		r	r	r	r	r	r	r	r	r	r	r	r
I felt embarrassment during the bowel preparation	0.081	0.103	0.157		0.163	0.378^a^	0.179	0.115	-0.305^a^	-0.209^a^	−0.202	−0.029	0.205	0.165	0.192	0.344^a^
I felt embarrassment when the colonoscope introduced^b^	0.162	0.144	0.179			0.276	0.199	0.15	−0.314^a^	−0.279^a^	−0.114	−0.019	0.184	0.199	0.268^a^	0.379^a^
I felt embarrassment throughout the colonoscopy^b^	0.145	0.242	0.191			0.350^a^	0.112	0.116	−0.390^a^	−0.358^a^	−0.074	−0.036	0.185	0.17	0.257	0.434^a^
I felt pain from during the bowel preparation	0.19	0.269	**0.315**^a^		0.189	0.186	0.126	0.226	−0.151	−0.099	−0.153	−0.083	0.159	0.196	0.226	0.213
I felt pain when the colonoscope introduced^b^	0.123	0.153	0.222			0.173	0.233	0.16	−0.255^a^	−0.183	−0.225^a^	−0.097	0.194	0.183	0.246	0.391^a^
I felt pain throughout the colonoscopy^b^	0.177	0.184	0.213			0.232	0.198	0.132	−0.218	−0.121	−0.304^a^	−0.136	0.171	0.193	0.261	0.268
After examination, I felt abdominal discomfort and pain	0.105	0.154	0.11		0.093	0.192	0.148	0.192	−0.274^a^	−0.234^a^	−0.083	−0.022	0.196	0.12	0.169	0.158
I felt burdened to drinking bowel cleansing agents (drinking too much)	0.254	0.157	**0.284**^a^		0.139	0.274	0.231	0.078	−0.271^a^	−0.230^a^	−0.035	−0.157	0.227	0.18	0.261^a^	0.297^a^
I felt burdened to drinking bowel cleansing agent (bad taste)	**0.349**^a^	0.054	**0.255**^a^		0.09	0.277	0.229	0.12	−0.383^a^	−0.336^a^	−0.043	−0.189	0.270^a^	0.156	0.191	0.307^a^
I felt burdened to drinking bowel cleansing agent (affecting sleep)	0.203	0.115	0.202		0.111	0.089	0.107	0.059	−0.08	−0.036	0.017	−0.163	0.171	0.161	0.176	0.309^a^
I felt stressed when the colonoscope introduced^b^	0.094	0.09	0.137			0.229	0.311	0.237	−0.074	−0.023	−0.108	−0.039	0.264	0.284^a^	0.203	0.380^a^
I felt stressed throughout the colonoscopy^b^	0.141	0.143	0.203			0.208	0.239	0.243	−0.171	−0.136	−0.044	−0.028	0.157	0.229	0.263	0.529^a^
I felt stressed when waiting for the final results	0.149	0.215	0.148		0.149	0.11	0.14	0.128	−0.053	−0.091	0.119	0.192	0.144	0.269^a^	0.154	0.281
After examination, I felt abdominal discomfort and stressed	0.198	0.138	0.124		0.125	0.069	0.23	0.114	−0.085	−0.072	−0.042	0.07	0.128	0.183	0.15	0.134

IBD, Inflammatory bowel disease; BMI, body mass index.

a : *P* < 0.05. ^b^: Patients who chose painless colonoscopy last time were not included in this item (*n* = 79).

### 3.3 Doctor-recommended and patient-acceptable intervals for colonoscopy

**Fig. 2:**
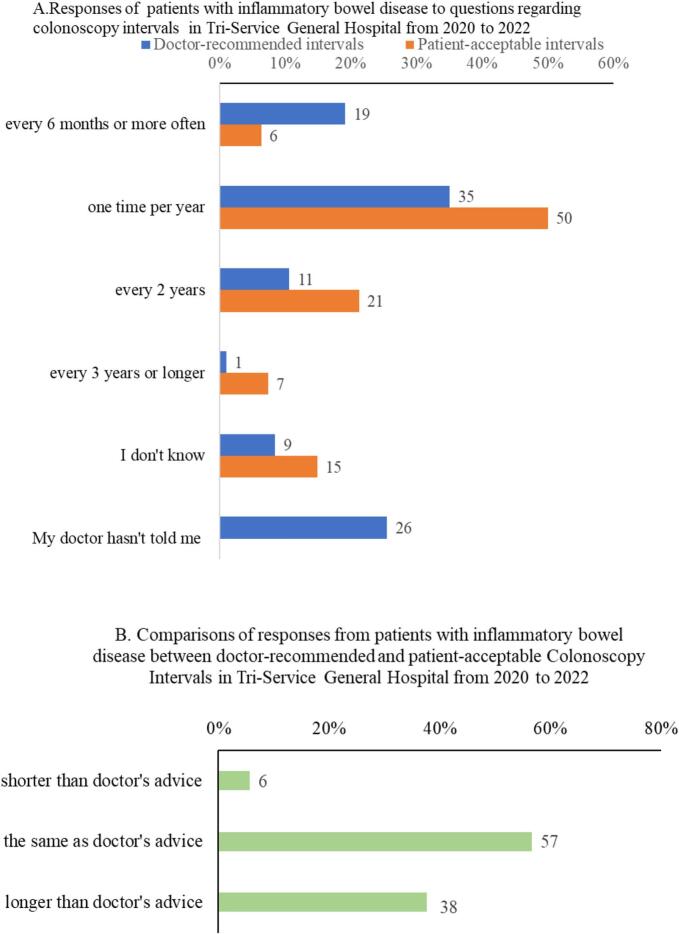
Frequency of doctor-recommended intervals compared with the frequency of patient-acceptable intervals for colonoscopies in patients with inflammatory bowel disease in Tri-Service General Hospital from 2020 to 2022.

The responses of patients to questions regarding colonoscopy intervals are shown in Fig. 2A. Our results showed significant differences between doctor-recommended and patient-acceptable examination intervals (*P* = 0.001), excluding responses categorized as “My doctor hasn't told me”. We also compared the doctor-recommended and patient-acceptable examination intervals, excluding patients who chose “I don't know” or “My doctor hasn't told me,” and the results are shown in Fig. 2B. Importantly, the acceptable examination interval for most patients (57 %) was consistent with the examination interval recommended by the doctor.

### 3.4 Correlation between each questionnaire item regarding colonoscopy interval and patient characteristics

Table 3 summarizes the correlation analysis between “Patient's acceptable examination interval” and patient characteristics. Patients of younger age and those diagnosed at an earlier age were more likely to prefer a short time interval between colonoscopies (correlation coefficients of 0.286 and 0.251, respectively). Patients who underwent colonoscopy without sedation showed similar results (correlation coefficients of 0.264 and 0.259, respectively). We further evaluated the correlation between the difference between the doctor-recommended interval and patient acceptance frequency and the characteristics of the patients. We found that age and age at diagnosis were significantly negatively correlated with the difference between the two intervals (correlation coefficients: −0.394 and − 0.343, respectively), excluding those who opted for sedated colonoscopy during the previous attempt. There was a notably positive correlation between prior experience of sedated colonoscopy and the difference between the two intervals. Among all patients, the preference for Western food and a history of undergoing sedated colonoscopy showed a significantly positive correlation with the difference between the patient-acceptable intervals and doctor-recommended intervals (correlation coefficients: −0.409 and 0.340, respectively).

**Table 3:** Correlation matrix of frequency of colonoscopy in patients with inflammatory bowel disease in Tri-Service General Hospital with important parameters from 2020 to 2022.

	Sex	IBD type	BMI grouping	Sedation given	Education level	Disease duration grouping	Previous bowel resection	Previous of sedated colonoscopy	Age	Age at diagnosis	Disease duration	BMI	Smoking	Alcohol	West food
	r	r	r	r	r	r	r	r	r	r	r	r	r	r	r
(include who chose sedated colonoscopy last time)															
bDoctor-recommended intervals	0.177	0.25	0.248	0.281	0.322	0.15	0.051	0.241	0.441^a^	0.334^a^	0.223	0.173	0.221	0.283	0.425^a^
cPatient-acceptable intervals	0.281	0.192	0.171	0.164	0.267	0.22	0.223	0.162	0.286^a^	0.251^a^	0.01	0.109	0.14	0.169	0.211
Patient-acceptable intervals versus doctor-recommended intervals	0.117	0.108	0.251	0.121	0.182	0.153	0.101	0.340^a^	−0.26	−0.146	−0.21	−0.067	0.147	0.326	0.409^a^
(exclude those who chose sedated colonoscopy last time)															
bDoctor-recommended intervals	0.075	0.255	0.301	N/A	0.276	0.2	0.161	0.292	0.453^a^	0.429^a^	0.129	0.251	0.267	0.295	0.358
cPatient-acceptable intervals	0.274	0.124	0.2	N/A	0.274	0.239	0.254	0.155	0.264^a^	0.259^a^	−0.051	0.167	0.134	0.172	0.195
Patient-acceptable intervals versus doctor-recommended intervals	0.193	0.161	0.253	N/A	0.114	0.121	0.09	0.380^a^	−0.394^a^	-0.343^a^	−0.103	−0.118	0.14	0.337	0.318

BMI, body mass index; IBD, Inflammatory bowel disease.

a : P < 0.05.

b : exclude “I don't know” and “My doctor hasn't told me.”

c : exclude “I don't know.”

## 4 Discussion

This study investigated patients' experiences and feelings during colonoscopy and bowel preparation and their attitudes toward the frequency of colonoscopy. The results showed that a significant proportion of patients experienced embarrassment, pain, discomfort, and stress during colonoscopy and bowel preparation, with bowel preparation and abdominal pain being the most unacceptable factors for patients undergoing colonoscopy.

This study identified factors affecting routine/surveillance colonoscopy compliance in patients with IBD, which can help promote adherence to colonoscopy and facilitate early detection of major complications in high-risk patients and those with long-term IBD. However, some limitations need to be acknowledged. First, this study only included patients with IBD, which may limit the generalizability of the findings to other patient populations. Second, the study relied on self-reported data, which may have been subject to recall or social desirability biases. For instance, long-term doctor-patient relationships were observed in patients with IBD. Thus, this reliance on self-reported data can be one aspect that would influence the results of our study, leading to biases. Third, the sample size was relatively small, which might have limited the statistical power of the study and its ability to detect significant differences. We look forward to strengthening hospital collaboration and transforming it into an inter-institutional research plan to expand sample size in further studies. Fourth, the study did not include a control group; thus, comparing the results to patients without IBD would be difficult. Finally, the study did not assess the impact of other factors, such as socioeconomic status or comorbidities, on patients' perception and acceptance of colonoscopy. Nonetheless, this study provides valuable novel insights into the attitudes and experiences of patients toward colonoscopy procedures and examination intervals.

The burden of bowel preparation remained the most prominent factor contributing to a negative attitude toward colonoscopy, particularly excessive fluid consumption. In another study, bowel preparation was the most burdensome aspect of the entire colonoscopy procedure (Denters et al., 2013a), especially in patients with IBD (Denters et al., 2013b). We also evaluated the association between various bowel preparation agents and feelings of discomfort. In our study, most patients used sodium sulfate combined with PEG as a bowel preparation agent, followed by sodium picosulfate combined with magnesium citrate. Sodium picosulfate was considered suitable for patients who could not tolerate a large volume of fluid (Bechtold et al., 2016). Bowel preparation is necessary for colonoscopy, especially for detecting dysplastic lesions and observing mucosal healing (Martens and Bisschops, 2014). Previous studies revealed that PEG-LS had a better effect, leading to significantly more adequate bowel preparation than sodium picosulfate (Rostom et al., 2019). However, our study observed no specific correlation between discomfort and the different bowel preparation agents. In our study, higher BMI was positively correlated with pain during the bowel preparation phase. Flatus is considered the cause of abdominal discomfort in patients with a higher BMI. In a previous study, patients with obesity (BMI >30 kg/m^2^) were more likely to have inadequate bowel preparation at colonoscopy (Laurie et al., 2022). Patients with obesity exhibit higher concentrations of inhibitory neuropeptide vasoactive intestinal polypeptide, which reduces colonic secretion and motility (Motomura et al., 2000). To the best of our knowledge, no previous study has revealed a positive correlation between higher BMI and pain during colon preparation in patients with IBD.

Approximately 25 % of patients who previously selected colonoscopy without sedation felt it was quite or very painful when the colonoscope was inserted and during the colonoscopy process. In our study, longer disease duration was negatively correlated with pain during colonoscope insertion and colonoscopy. This finding contradicts the previous literature. Longer disease duration indicates a longer colonoscopy follow-up period. A previous study revealed that patients with IBD who had experienced earlier painful colonoscopies were more worried about pain and were more likely to experience severe pain during the examination (Ryhlander et al., 2019). One possible explanation for this discrepancy is that the colonoscopies in our patients with IBD were performed by endoscopists who specialized in IBD. In addition, a previous study reported that the patients' bond of trust with their treating physician was the most important factor for IBD patients (Denters et al., 2013a). In the cohort, patients' age and age at diagnosis were negatively correlated with the feeling of abdominal discomfort and pain after examination. Reportedly, carbon dioxide insufflation can help manage post-colonoscopy pain (Cleland et al., 2013; Falt et al., 2017). However, all patients in our study received carbon dioxide insufflation during colonoscopy. Inflammatory bowel status is a possible precipitating factor for post-colonoscopy abdominal pain. Colonoscopy with sedation should be considered for such patients, but good disease control is also an important factor.

We observed that longer intervals between colonoscopies were recommended for older patients with IBD and patients with IBD diagnosed at an older age, as well as patients who had previously selected colonoscopy without sedation. The difference between doctor-recommended and patient-acceptable intervals is an important point in our study. Approximately half of the patients (57 %) agreed with the examination interval recommended by the doctor; however, 38 % preferred longer examination intervals. Younger patients who underwent colonoscopy without sedation tended to prefer longer examination intervals than older patient groups. Therefore, colonoscopy with sedation was suggested for younger patients to improve adherence to the colonoscopy schedule recommended by the physician. In patients with IBD, a prior experience of sedated colonoscopy was positively correlated with the variance between the patient-acceptable intervals and the doctor-recommended intervals.

The strength of our study is that it is the first study on the perception of colonoscopy and attitude toward the examination interval in Asian patients with IBD. Different clinical phenotypes of diseases and real-world clinical practices for IBD management have been reported between Western and Asian countries (Clarke and Feuerstein, 2019; National Institute for Health and Care Excellence: Guidelines, 2022; Wei et al., 2017a; Wei et al., 2017b; Ran et al., 2021). Our questionnaires focused on patients' feelings about bowel preparation and the colonoscopy process. A previous study found that pre-procedural nervousness strongly predicted an adverse endoscopic experience (Umezawa et al., 2015). Furthermore, economic issues may impact adherence to colonoscopy (Matthew et al., 2020). Expenses of colonoscopy differ in each country. The National Health Insurance Service (NHI) covers most colonoscopy costs in Taiwan. Extra expenses would be incurred if the patient requested a sedated colonoscopy. It is possible that economic issues may not have an impact on Taiwanese patients, but further research is needed to confirm this. We also analyzed education level as a parameter affecting patients' perception and acceptance of colonoscopy. The analysis revealed a positive relationship with “felt embarrassment during the bowel preparation” and “felt embarrassment throughout the colonoscopy”. However, it did not reveal a positive relationship with the issue of patient-acceptable intervals. In our study, approximately 32 % of patients were not familiar with the doctor-recommended interval; this proportion is considerably higher than that reported in a previous study (Friedman et al., 2013). An expert report on IBD in Asians stated that colonoscopy is not properly performed for dysplasia/cancer surveillance and post-operative recurrence of Crohn's disease in Asian countries (Ahuja V Ahuja et al., 2024). It also revealed that while most countries initiate surveillance colonoscopy within 10 years from disease onset, the median time from disease onset to surveillance initiation was over 10 years in some countries. This finding highlights that a physician's professional practices may impact patient prognosis due to the failure to properly schedule colonoscopies for patients with IBD and educate them on the importance of routine surveillance in Asia.

This study provides insights into patient perceptions and attitudes toward colonoscopy, bowel preparation, and examination intervals. Bowel cleansing and abdominal pain were the most uncomfortable aspects of the colonoscopy procedure in Asian patients with IBD, especially without sedation. A 1-year interval was the most acceptable option for colonoscopy scheduling in patients with IBD. Younger age and younger age at diagnosis would impose a greater burden on bowel preparation and pain after colonoscopy. Patients with a longer disease duration tended to have less pain during colonoscopy. Therefore, the use of sedation during colonoscopy could be an alternative option for subgroups of patients with IBD, especially younger patients diagnosed with IBD. This study provides important information on IBD patients' perceptions of colonoscopy that would enhance adherence to colonoscopy and increase early detection rates of dysplastic lesions or colorectal cancer in high-risk and long-term IBD patients.

## Funding

This work was supported by the Medical Affairs Bureau, 10.13039/501100003559Ministry of National Defense [grant number MND-MAB-D-111114], 10.13039/501100010425Tri-Service General Hospital [grant number TSGH-E-110192], and National Science and Technology Council, Taiwan [grant number 111–2314-B − 016 -048].

## Informed consent

All patients provided written informed consent before study inclusion.

## Ethics statement

The study was approved by the Institutional Review Board of the Tri-Service General Hospital (approval no. B202005094).

## Prior presentations

Part of this study was presented at the 11th annual meeting of the Asian Association for Crohn's and Colitis (AOCC), 2023.

## CRediT authorship contribution statement

**Chang-Hung Liao:** Writing – review & editing, Writing – original draft, Software, Methodology, Formal analysis, Data curation. **Peng-Jen Chen:** Validation, Supervision. **Yu-Lueng Shih:** Visualization, Supervision. **Wei-Kuo Chang:** Supervision. **Tsai-Yuan Hsieh:** Supervision. **Tien-Yu Huang:** Writing – review & editing, Resources, Project administration, Investigation, Funding acquisition, Conceptualization.

## Declaration of competing interest

The authors declare that they have no known competing financial interests or personal relationships that could have appeared to influence the work reported in this paper.

## Data Availability

The datasets used and/or analyzed during the current study are available from the corresponding author upon reasonable request.
